# Identifying factors influencing emerging innovations in hospital discharge decision making in response to system stress: a qualitative study

**DOI:** 10.1186/s12913-024-11784-5

**Published:** 2024-10-28

**Authors:** Allison M. Gustavson, Matthew J. Miller, Natassia Boening, Emily M. Hudson, Jennifer P. Wisdom, Robert E. Burke, Hildi J. Hagedorn

**Affiliations:** 1 Veterans Affairs Health Services Research and Development Center for Care Delivery and Outcomes Research, Minneapolis Veterans Affairs Health Care System, Minneapolis, MN 55417 USA; 2 Department of Medicine, University of Minnesota, Minneapolis, MN 55455 USA; 3 Department of Physical Therapy and Rehabilitation Science, University of California, San Francisco, San Francisco, CA USA; 4 Wisdom Consulting, New York, New York, NY 10023 USA; 5 Center for Health Equity Research and Promotion, Corporal Crescenz VA Medical Center, Philadelphia, PA USA; 6 Division of General Internal Medicine, University of Pennsylvania Perelman School of Medicine, Philadelphia, PA USA; 7 Leonard Davis Institute of Health Economics, University of Pennsylvania, Philadelphia, PA USA; 8 Department of Psychiatry, University of Minnesota School of Medicine, Minneapolis, MN 55455 USA

**Keywords:** COVID-19 pandemic, Learning Health System, Transitions of care, Veterans patient discharge, Decision making

## Abstract

**Background:**

The purpose of this qualitative study was to identify emergent rehabilitation innovations and clinician perceptions influencing their implementation and outcomes related to hospital discharge decision-making during the Coronavirus 2019 pandemic.

**Methods:**

Rehabilitation clinicians were recruited from the Veterans Affairs Health Care System and participated in individual semi-structured interviews guided by the integrated Promoting Action on Research Implementation in Health Services (i-PARIHS) framework. Data were analyzed using a rapid qualitative, deductive team-based approach informed by directed content analysis.

**Results:**

Twenty-three rehabilitation clinicians representing physical (*N* = 11) and occupational therapy (*N* = 12) participated in the study. Three primary themes were generated: (1) Innovation: emerging innovations in discharge processes included perceived increases in team collaboration, shifts in caseload prioritization, and alternative options for post-acute care. (2) Recipients: innovations emerged as approaches to communicating discharge recommendations changed (in-person to virtual) and strong patient/family preferences to discharge to the home challenged collaborative goal setting; and (3) Context: the ability of rehabilitation clinicians to innovate and the form of innovations were influenced by the broader hospital system, interdisciplinary team dynamics, and policy fluctuations. Innovations described by participants included (1) use of technological modalities for interdisciplinary collaboration, (2) expansion of telehealth modalities to deliver care in the home, (3) changes in acute care case prioritization, and (4) alternative options for discharge directly to home.

**Conclusions:**

Our findings reinforce that rehabilitation clinicians developed innovative strategies to quickly adapt to multiple systems-level factors that were changing in the face of the COVID-19 pandemic. Future research is needed to assess the impact of innovations, remediate unintended consequences, and evaluate the implementation of promising innovations to respond to emerging healthcare delivery needs more rapidly.

**Supplementary Information:**

The online version contains supplementary material available at 10.1186/s12913-024-11784-5.

## Background

Rehabilitation clinicians’ discharge recommendations are one of the strongest predictors of hospital readmissions and other adverse events [1–3]. After COVID-19, discharge decision-making fundamentally changed as patients sought to avoid post-acute care facilities by discharging to home. Furthermore, the ability to provide necessary care in the home was hampered by ongoing, COVID-19 exacerbated industry challenges including an undervalued workforce and insufficient staffing [4–7]. Yet, we do not have a deeper understanding of *how* rehabilitation clinicians innovate during their discharge decision making processes in the face of external pressures to the health care system. This information has the potential to generate hypotheses to test changes to models of transitional care and inform measurement of unintended consequences (e.g., costly rehospitalizations or multiple, unnecessary transitions in care).

Given the rapid and evolving nature of the COVID-19 pandemic and post-pandemic procedures, a Learning Health System (LHS) approach is needed to study how pandemic-induced changes influenced the emergence of innovation in rehabilitation clinicians’ approaches and recommendations for hospital discharge. The LHS approach encourages continual learning between clinical practice and research through a bi-directional cycle of data to knowledge, knowledge to practice, and practice to data [8–10]. Learning Health Systems use rapid cycle methods that integrate data and real-world experiences to iteratively evaluate clinical practices and models of care [11–15]. The Veterans Health Administration (VHA) is the largest integrated health system in the United States and has communicated a vision to adopt LHS approaches into all aspects of research, clinical care, education, and emergency preparedness to optimize care and outcomes for over 9 million veterans served [12, 13, 16]. Thus, we wanted to explore VHA rehabilitation clinicians’ perceptions of hospital discharge decision making and transitions of care to identify emerging innovations that could inform future work evaluating the impact of promising innovations and spread or scale-up.

The continuous learning cycle supported through a LHS lens allows us to translate our approach to identifying and exploring emerging innovations—across a variety of clinical disciplines, settings, and systems–closer to real-time, thus allowing for more rapid and informed responses to system stressors. The COVID-19 pandemic is but one example of an external pressure and innovations in discharge decision-making are likely to occur again, particularly in the face of technological advances and possible post-pandemic reforms in healthcare. With our current focus on hospital discharge, understanding organic innovation is a critical element to move forward with research and quality improvement initiatives to ensure safe, cost-effective, and patient-centered care transitions during the pandemic and beyond. Therefore, the aim of this qualitative study was to identify emergent rehabilitation innovations and clinician perceptions influencing their implementation and outcomes related to hospital discharge decision-making during the COVID-19 pandemic. Our study’s overarching focus was on the data to knowledge aspect of LHS, by which we used qualitative approaches to identify emerging innovations and perceptions of best practices in hospital discharge recommendations and transitions of care. Results were rapidly disseminated to key leadership to inform future evidence and implementation of knowledge to practice.

## Methods

### Study design

We used a qualitative study design guided by the integrated Promoting Action on Research Implementation in Health Services (i-PARIHS) conceptual framework [17, 18]. The i-PARIHS model has been adapted previously to address complex implementation science research questions, thus we adapted i-PARIHS to identify organic innovations (e.g., practice, intervention, approach) around discharge decision making (data to knowledge) rather than to evaluate implementation of a specific intervention (knowledge to practice). While i-PARIHS considers facilitation a central component to aligning the innovation, recipients, and context for successful implementation, instead, we examined the interactions among the potential clinical innovations, recipients involved, and contextual factors. Using i-PARIHS in this way provides the foundation for facilitation to be used when considering implementation of emerging innovations that occur in response to system stressors. In i-PARIHS *innovation* is traditionally defined as the intervention characteristics, including the complexity of the intervention, relative advantage compared to current practice or another intervention, usability, and evidence to support the intervention (e.g., research-based evidence, clinical experience, and patient preferences). In this study, *innovation* consisted of practices or approaches that organically emerged during the COVID-19 pandemic to manage hospital discharge decision-making and processes [17, 18]. The i-PARIHS construct *recipients* includes characteristics of the people who are affected by the innovation including patients and care partners [17, 18]. *Context* includes aspects internal to the organizational setting including leadership support, culture, organizational priorities, evaluation and feedback processes, and learning networks. *Context* also includes aspects external to the organization, such as policy drivers and priorities, incentives and mandates, and inter-organizational networks [17, 18]. 

### Participants

We used convenience sampling to recruit occupational therapists (OTs), occupational therapist assistants (OTAs), physical therapists (PTs), and physical therapist assistants (PTAs) who spend at least 25% of their time providing acute or post-acute care service in the Veterans Affairs (VA) Health Care System. An initial recruitment letter was sent out to rehabilitation clinicians in the Minneapolis VA Health Care System (MVAHCS), where co-authors have a relationship with clinical leadership. We then used a snowball technique [19, 20] to supplement our recruitment efforts by expanding beyond the MVAHCS to other VAs. Informed consent was obtained prior to data collection, which included an overview of the purpose of the study, risks and benefits to participating, confidentiality, and the voluntary nature of the study.

### Data collection

Qualitative data were collected from rehabilitation clinicians using 30–60 min semi-structured interviews conducted between May 2022 and August 2022. Interviews were conducted virtually. Interview participants self-reported descriptive characteristics that included discipline and years of practice. Union rules prohibited collection of any other demographic information (e.g., sex, race/ethnicity, age, highest degree earned). The interview guide (Table 1) was developed through an iterative, team-based approach and informed by expert input from key informants at the MVAHCS (two of whom participated in the interviews), a review of relevant literature [21–23], and the i-PARIHS framework [17, 18]. Semi-structured interview procedures included a welcome, introduction, study purpose, interview goals, and a statement of confidentiality. The goal of the interview was for rehabilitation clinicians to provide detailed descriptions of their discharge decision making process and perceptions of the patient’s transition from hospital to home, which could then be further analyzed within the i-PARIHS framework. VA union rules prohibited recording and transcribing qualitative interviews with the rehabilitation clinicians participating in this study. To ensure trustworthiness of data, interviews were documented using extensive field notes by trained research members [24]. During the interviews, two members of the research team were present to document the interviews through extensive field notes. These two members of the research team met immediately after the interview to collaboratively review the field notes, revise notes for accuracy of participant comments, and document immediate impressions. To further reduce risk of bias, we also conducted member checking [19, 20] as described below in the section titled Reflexivity and rigor.

**Table 1:** Semi-structured interview guide

1. **Please tell me about your experience making discharge recommendations for patients in the COVID-19 era (i.e., currently)**.
Probes: a. Can you describe your process for developing discharge recommendations for a specific patient for home discharge or a complex home discharge? b. How did you ensure patient safety for discharge? c. How did you meet patient/family needs? d. How did you meet your own needs and that of your team’s? e. How did you meet healthcare system needs? f. What resources did you utilize (or want to utilize) to guide discharge recommendations? g. What resources would you need to spread this process/idea within your facility or to other facilities?
2. **How is this discharge decision making differed from pre-COVID?**
Probes: a. What changes could have made the experience better for you? b. What changes could have made the experience better for patients and/or family? c. What changes could have made the experience better for the healthcare system? d. What could make the transition from pre-COVID to COVID discharge recommendations better? a. What resources would you need to spread this change in process/idea within your facility or to other facilities?

### Data analysis

We used a rapid qualitative, deductive team-based approach [25, 26] informed by directed content analysis [27, 28]. Aligned with the LHS approach [8], a rapid qualitative approach [25, 26] was employed as the healthcare system continues to have evolving processes post-pandemic and the insights gathered are timely and pertinent. Insights were disseminated to key leadership throughout the data analysis process to provide immediate value to the MVAHCS, such as influencing the content and utility of research in this area as well as by informing decision making in real-time regarding potential resource needs and promising approaches that could be scaled quickly to address ongoing system capacity challenges. Directed content analysis uses a predefined framework (in this case i-PARIHS [17, 18]) to guide data collection, analysis, and interpretation [28]. 

We used the constructs outlined in i-PARIHS (*recipients*, *context*, and *innovation*) to guide deductive themes and were also open to emergent themes. For analysis, we compiled extensive field notes into a matrix for analysis (Microsoft Excel). The rows consisted of participants and columns represented the pre-identified categories mapped to the interview guide questions. Once all data was organized for each participant, the data was transferred to a word document (Microsoft Word) where repetitive data were removed. Three members of the research team (AMG, MJM and JPW) met bi-weekly to iteratively discuss and analyze the accruing data by deductively categorizing the raw data into predefined themes by timepoint (pre-pandemic versus COVID-19 era) and discussing the potential for emerging themes or the need to collapse themes. During this time, the team also identified representative field note excerpts for emergent themes. This team-based approach to coding allowed a less arduous, yet still rigorous, approach to ensuring data dependability and trustworthiness compared to calculating inter-rater reliability [29]. Finally, the multi-disciplinary research team met bi-weekly to discuss resulting themes and how they compared or contrasted across timepoints. Saturation was defined by the absence of new and emerging themes within the data [30]. 

### Reflexivity and rigor

This qualitative study followed the consolidated criteria for reporting qualitative research checklist (COREQ) to ensure the rigor of the study design, conduct, and interpretation of findings [31]. Prior to conducting any interviews, researchers (HJH, AMG, NB) were extensively trained by co-author JPW—including completing practice interviews–to ensure consistency with qualitative interview processes. All members of the research team involved in the interview process received extensive training by co-author JPW on documenting field notes using practice interviews and corroborated notes for accuracy [24]. We also used a team-based approach to open coding to create inter-coder consensus [29]. As a form of member checking, we discussed a draft table of results with three participants to ensure trustworthiness of our rapid qualitative analysis of findings [19, 20, 32]. During this member check, participants provided feedback on near completed qualitative findings, voiced agreement or disagreement, as well as provided additional context for our findings. These data were then used to finalize our qualitative results.

### Role of funding source

The funders played no role in the design, conduct, of reporting of this study.

## Results

We collected data from 23 rehabilitation clinicians (PT/PTA: 11; OT: 12) across 10 VHA facilities. No OTAs were recruited. The mean number of years working as a rehabilitation clinician was 13.7 (SD = 6.4; range 5–24 years) with the mean number of years working in the VHA system at 11.3 (SD = 6.7; range 1–23 years). For each of the i-PARIHS constructs, we identified primary themes emerging from the qualitative data. The primary themes described emergent *innovations* and factors that influence the emergence of these innovations (*context* and *recipients*). The primary theme for *innovation* was “evolving processes.” The primary theme for *recipients* was “patient & care partner needs.” The primary theme for *context* was “system & personnel needs”. Table 2 outlines exemplar excerpts from our field notes.

**Table 2:** i-PARIHS constructs and emergent themes by pre-pandemic and COVID-19 era time frames with exemplar field note summaries

i-PARIHS Construct and Emergent Themes	Pre-Pandemic	COVID-19 Era
**RECIPIENTS: Patient & Care Partner Needs**		
Planning & Communication	• In-person communication to understand preferences for providing care, level of care needed, safety concerns, confidence, and additional services • Communicate interdisciplinary goals for short-term rehabilitation to increase planning time for post-hospital to home discharge	• Phone or video communication with care partner regarding patient needs and whether the patient returned to functional baseline • Visitor restrictions make it challenging to provide care partners hands-on training and planning for safe discharge
Complex Nature of Needs	• Collaborative goal setting is important to understanding and remediating conflicting patient *and* care partners’ needs • Final discharge decisions come down to insurance coverage or environmental barriers • Repeat admissions may signal unmet needs	• Higher reliance on under-staffed home health care & services because of patient or care partner preference to discharge home meant unmet needs may be present prior to hospitalization and persist post-discharge with additional gaps in new needs
**CONTEXT: System & Personnel Needs**		
System Influence on Clinician Practice	• System pressure to get patients out of the hospital and, for most, to sub-acute care for rehabilitation • Documentation is critical to creating a narrative and justification for discharge decisions to ensure reimbursement for the necessary level of care is provided	• Rehabilitation clinicians initially operated under the assumption that patients need to discharge home as opposed to short-term rehabilitation facilities though tension existed when considering home health care & services quality and staffing shortages
Team Role of Rehabilitation Clinicians	• Rehabilitation clinicians typically invited to join in the discharge planning and decisions late in hospital course • Last line of defense to sending a patient home in an unsafe situation • Provide education for role of rehabilitation team in interdisciplinary assessment and discharge planning	• Role shift from primarily acute assessment model to sub-acute care model to address prolonged hospital lengths of stay and limited or unavailable post-acute resources • Rehabilitation role in devising plans of care (frequency of acute services) for patients with longer hospital stays received more scrutiny from higher levels of leadership
Pandemic-Related Fluctuations in Practices, Processes, & Policies	N/A	• High stress and emotional burden experienced with ever changing practice, processes, and policies • Rehabilitation team is not heard or given the chance to adjust to evolving changes in the acute care context
**INNOVATION: Evolving Processes**		
Interdisciplinary Collaboration	• High-level of clinical reasoning and understanding of factors in the context of the patient’s individual factors, social supports, & environmental constraints	• Improved team communication and collaboration through virtual communication platforms
Prioritization of Workflow & Caseloads	• Imminent discharge sessions/visits were prioritized • Rehabilitation clinicians build-in follow-up visits due to the natural progression of recovery and the potential need to update discharge recommendations accordingly	• Earlier initiation of discharge planning and decision-making for complex patients with difficult discharge placements that prolong hospital length of stay • Adjust plans of care (number of visits per week) to adapt to longer hospital stays while balancing the need to see more acute patients
Reducing Readmission Risk	• Recommendation default is sub-acute care for rehabilitation • Multiple hospitalizations trigger conversation and reflection on what previous discharge recommendations worked and did not and why • Care partner support and home rehabilitation is essential for safe discharge to home	• Shift towards recommendations to home with supports and services • Identify and consider alternative approaches to assisting patients who are “failing at home” and admitted to the hospital frequently • Wider array of discharge options that balance tensions and strains of the pandemic on health and social supports • Teleworking has increased potential to conduct follow-up calls

### Innovation: evolving processes

The *innovation* construct of i-PARIHS was portrayed by the primary theme of evolving processes that emerged with the onset of the COVID-19 pandemic. Participants described different approaches to hospital discharge decision making pre-pandemic versus the COVID-19 era, with noted innovations occurring because of contextual and recipient factors arising from the COVID-19 era. Emergent, nested sub-themes included interdisciplinary collaboration; prioritization of workflow and caseloads; and reducing readmission risk. Innovations described by participants included (1) use of technological modalities for interdisciplinary communication and collaboration regarding discharge decision making, (2) expansion of telehealth modalities to deliver care to patients in the post-acute period that influenced *where* patients could safely discharge, (3) changes in acute care case prioritization, and (4) alternative options for discharge directly to home.

#### Interdisciplinary collaboration

Pre-pandemic, participants described the complex decision-making process that requires a high level of clinical reasoning to sift through the interaction between a patient’s individual (e.g., falls risk, cognition, health literacy), social (e.g., caregiver support), and environmental (e.g., home environment, transportation) factors. Interdisciplinary collaboration was stated as a key component to making discharge recommendations. During the COVID-19 era, most participants talked about challenges with communication between interdisciplinary team members due to limited in-person interactions. As a result, participants talked about innovations in improved inter- and intra-disciplinary communication and collaboration through the expanded capacity of virtual communication platforms (e.g., Microsoft Teams, virtual care platform) that were newly implemented in the federal system. For example, one participant explained that despite limited face-to-face interactions, chat and calling features on a secure network allowed for faster response time and virtual hand-offs. The absence of family or care partners to confirm or provide accurate information to inform discharge process and decisions was a significant challenge to meeting patient and family needs. As a result, during the COVID-era most participants described innovations related to enhanced interdisciplinary team coordination (formal and informal) that occurred to make sure everyone was on the same page in terms of what information is provided (by patients, family, or care partners) and how it impacts the discharge decision. Participants spoke about how the pandemic-accelerated expansion of virtual care options allowed clinicians to make discharge decisions that would ensure patients would receive the necessary follow-up care and support services. Participants described the continuity of care that could be provided by the VA when VA physical and occupational therapists could provide virtual care to veterans in their home following hospitalization.

#### Prioritization of workflow & caseloads

Pre-pandemic, most participants described workflows where patients with imminent hospital discharges were prioritized to receive visits or services versus those awaiting placement and needing a sub-acute level of care. After high-priority cases were seen, participants talked about how their built-in workflows allowed for follow-up to capture the natural progression of recovery and potential changes in functional status that could influence updates to discharge recommendations. Participants spoke about how follow-up allowed for reassessment of the patient’s progress and function to determine what level of sub-acute rehabilitation care may (or may not) be needed and the most appropriate discharge location. For patients where a discharge directly to home was considered, participants indicated caregiver presence was crucial along with the willingness to complete home physical or occupational therapy to maximize function and ability to complete activities of daily living.

During the COVID-19 era, some participants spoke about innovations related to earlier discharge planning and decision-making for more complex patients for whom discharge placement would be difficult. They acknowledged that patients with difficult placements were not a new challenge, but the pandemic has made such placements and the increased hospital lengths of stay more visible in an era where hospital stays may be longer due to limited or unavailable post-acute resources. When discussing the COVID-19 era, participants described adjusting the frequency of services per week in two scenarios. The first scenario was a sub-acute model of care where they were providing treatment 5 days per week to patients who would be going home, and pre-pandemic would have discharged to sub-acute care. For example, participants described pre-pandemic plans of care for patients waiting for sub-acute care as 1–3 days per week. In the second scenario, participants described decreasing plans of care (visits per week) in patients waiting for sub-acute care to get to more acute patients.

#### Reducing readmission risk

Pre-pandemic, most participants talked about the default recommendation being sub-acute rehabilitation to ensure the patient’s overall daily function was maximized prior to return home. Some participants indicated that multiple hospitalizations or recent readmissions signaled a need for further conversation with the patient and interdisciplinary team to reflect on what worked and did not work in the previous discharge plan. Participants spoke about the need for care partner support and willingness to complete home rehabilitation as an essential component of discharge making when considering a safe discharge to home.

Many participants noted a shift during the COVID-19 era towards more discharge recommendations to home with supports and services. This shift has varied throughout the pandemic due to restrictions in rehabilitation facilities being lifted and the availability of vaccines. For example, some participants described maintenance of this type of mindset to promote aging in place, while also indicating that other members of their team have slipped back to the previous default of discharge to sub-acute rehabilitation. Some participants indicated their discharge decision-making included more “thinking outside the box.” Participants described a wider array of discharge options that balanced tensions and strains of the pandemic on health and social supports. For example, one participant talked about how pre-pandemic the discharge recommendations were limited to 3–4 options (home, home with home health rehabilitation, sub-acute rehabilitation, and acute rehabilitation unit). Now in the COVID-19 era, the participant indicated more rehabilitation recommendation options were created to innovatively expand safe discharge options for home (e.g., home with durable medical equipment, home with skilled or unskilled care). However, some participants noted that patients may “fall through the cracks” due to the lack of follow-up to ensure equipment was received, home services and supports were started in a timely manner, and rehabilitation was initiated.

During the pandemic, participants indicated teleworking allowed time to reach out to patient’s following hospital or post-acute discharge to ensure equipment and home services were in place and assess whether the patient or care partner had additional questions. One participant noted that during the COVID-19 era, supply chain issues for durable medical equipment (e.g., standard wheelchair, lifts) have led to creative solutions to ensure patients have what they need as an alternative “bridge” to the equipment for safe discharge home. For example, rehabilitation clinicians leveraged equipment that might be available in satellite clinics to ensure patients were able to get critical needs, such as a walker or crutches, before discharging home. Another example was increased communication and collaboration with family—outside of the primary care partner—on a plan to safely assist in the home until lift equipment arrived.

### Recipients: patient & care partner needs

The *recipients* construct of i-PARIHS was encapsulated by the primary theme of patient and care partner needs. This theme relates to the receipt of and potential participation in hospital discharge decision-making by patients and their care partners. Two nested sub-themes, representing differing approaches to meeting the diverse, modifiable (or not), and potentially independent needs of patients and care partners included planning and communication and the complex nature of needs.

#### Planning & communication

Pre-pandemic, interviewees said they relied on in-person communication with care partners to understand a patient’s social support system including care partner availability, safety concerns, and additional services already in place. For instance, some participants spoke about in-person communication with care partners allowing for discussion of their preferences for and confidence in providing recommended care to the patient following hospital discharge. Participants then integrated this information with the patient’s functional ability and environmental factors to formulate a safe discharge recommendation that met both patient and care partner needs. Pre-pandemic, participants described communicating typical recommendations for sub-acute rehabilitation stay (e.g., skilled nursing facility) following hospitalization to patients and care partners to allow increased time for the interdisciplinary team, patient, and family to adequately plan for appropriate and safe discharge. During the COVID-19 era, participants maintained that communication with care partners remained an essential component of care despite difficulty communicating and providing hands-on training with care partners due to visitor restrictions. As such, communication with family or care partner occurred via phone or video which participants indicated may lead to miscommunications and misunderstanding of a patient’s current level of function and corresponding needs. A few participants talked about how the family needed to see the patient’s challenges in-person to fully understand what the patient’s needs would be at hospital discharge by comparing to his or her prior level of function. Additionally, visitor restrictions made hands-on training and subsequent planning for safe discharge challenging. One participant described providing care partner training over the phone and indicated it was ineffective because the clinician was unable to model the training (e.g., where to stand, how the clinician might assist with verbal cues, home set-up modifications).

#### Complex nature of needs

Pre-pandemic, participants described the active engagement of patients and care partners in collaborative goal setting to support autonomy in both the patient’s ultimate decision to go with or against discharge recommendations and the potential need for a higher level of care after hospital discharge (e.g., memory care or long-term care placement). In some complex situations, patient autonomy was influenced by insurance coverage or environmental needs. For example, one participant described an instance where a patient’s discharge options were limited due to experiencing homelessness, leading to an unsafe environment. When talking about the COVID-19 era, participants indicated that patient preferences to discharge home meant reliance on home health care and services that were experiencing staffing shortages. Participants suggested such staff shortages meant patient may have unmet needs prior to hospitalization that persist following discharge with added gaps in care and services to meet any new needs arising from recent hospitalization.

### Context: system & personnel needs

The *context* construct of i-PARIHS was captured by the primary theme of system and personnel needs. This theme relates to the environment in which the health care system and rehabilitation clinicians operated in to make and enact discharge planning and decisions. Participants described different contextual factors influencing hospital discharge decision making pre-pandemic versus the COVID-19 era, thus emergent sub-themes included system influence on clinician practice, the team role of rehabilitation clinicians, and pandemic-related fluctuations in practices, processes, and policies. Practices, processes, and policies generated in response to the pandemic emerged as a subtheme that was unique to the COVID-19 era.

#### System influence on clinician practice

Pre-pandemic, participants cited pressure to discharge patients to the next level of sub-acute care for rehabilitation to increase the time needed to plan for a safe discharge home. Participants talked about documentation as being essential to justifying rehabilitation clinicians’ decisions surrounding appropriateness for further rehabilitation (acute or sub-acute), the need for additional services, and recommendations to ensure the safest discharge possible. During the COVID-19 era—specifically in the initial stages of the pandemic–some participants indicated they were operating under the assumption that patients need to discharge home, even in cases where they would have recommended short term rehabilitation for the same patient in a pre-pandemic scenario. This perceived pressure to discharge to home during the COVID-19 era created tension as clinicians grappled with the current state of post-acute care. For example, one clinician explained that staffing shortages impacted home health care quality and timeliness, resulting in limited options for discharge.

#### Team role of rehabilitation clinicians

Some participants felt that members of the rehabilitation team were often the last ones invited to join in the discharge planning and decision-making process. These last-minute requests for assessment often left rehabilitation clinicians scrambling to set up home safety evaluations and care partner training. In some cases, participants felt their recommendations were the only thing keeping a patient from discharging to situation that was unsafe or where needs were not going to be met and readmission to the hospital was inevitable. For example, one participant explained that they were the “last defense” when advocating for a patient they deemed as not ready to discharge to home due to limited awareness for safety with transfers. The clinician explained that they worked with the family to provide training on transfers and evaluating assistive devices prior to discharge. Participants described delivering continuing education to interdisciplinary clinicians to address late involvement of rehabilitation clinicians and promote early and appropriate involvement of the rehabilitation team. Participants described their pre-pandemic role as primarily acute care assessment. During the COVID-19 era, some participants talked about a shift from their pre-pandemic assessment role to a sub-acute model of care—termed “Rehab in Place.” “Rehab in Place” was described as daily treatment while hospitalized with a discharge goal directed at home. Some participants reported that this shift created tension in work roles as workflow (e.g., triaging) and resources (e.g., staffing to provide treatments 6 to 7 days per week) needed to change to adequately support such a paradigm shift. Participants talked about how the COVID-19 pandemic initially made discharge to rehabilitation facilities difficult due to outbreaks and patient/family fears of contracting the virus at a communal facility. According to some participants, these difficult placements led to longer hospitalizations and adjustments to plans of care (i.e., frequency of acute services) provided by rehabilitation clinicians. As a result of these longer hospitalizations, some clinicians perceived elevated scrutiny from higher levels of leadership to either “Rehab in Place” with a higher frequency of services or develop alternative options for discharge.

#### Pandemic-related fluctuations in practices, processes, and policies

During the COVID-19 era, participants reported high stress and emotional burden due to various communication breakdowns regarding visitor policies, personal protective equipment policies, outbreak status, and changing public health recommendations. For example, one participant explained that they were tired and stressed due to the disconnect between management, leadership, and clinicians as the policies changed frequently and different plans were communicated from various leadership. While these changes in practice, processes, and policies have evolved with the pandemic, some participants felt rehabilitation teams were "not given the chance to breathe” and adjust to contextual changes in acute care delivery. Some participants spoke about the changes to workflow and resources as sources of tension between clinicians and organizational leadership.

## Discussion

This qualitative study found factors that influenced the emergence of innovation in hospital discharge decision-making during the COVID-19 era, thereby informing the data to knowledge aspect of LHS. Factors at the recipient and contextual level brought forth innovation to hospital discharge decision making. Emerging innovations, as perceived and described by rehabilitation clinicians, included interdisciplinary acute team collaboration, shifts in caseload prioritization, and alternative options for post-acute care. Our study adds to the literature by depicting a health care system’s response and emerging innovation in discharge decision making that fostered creative solutions to existing, new, and evolving issues with hospital discharge during the COVID-19 era.

We identified similar challenges during the COVID-19 era as those described by other qualitative studies [33, 34] including reports of reduced communication between clinicians and care partners, a focus on decreasing hospital lengths of stay, patients opting to go home rather than sub-acute rehabilitation, and ongoing stress and emotional burden with ever changing policies/practices. Our findings are also consistent with experiences of other healthcare professionals during the pandemic [35, 36]. Other qualitative studies of frontline providers during the pandemic described positive experiences that emerged from the challenges such as feeling part of something bigger than themselves and being valued as healthcare professionals [36, 37]. These experiences align with our findings of innovation in the midst of a systemic stressor. Importantly, the COVID-19 pandemic has drastically transformed health care, particularly with the accelerated integration of technology into care delivery [38, 39]. Thus, the current study has relevance in the post-pandemic world as we grapple with the many healthcare issues unmasked by the pandemic and seize the opportunity to leverage this momentum for healthcare transformation to reshape our approaches to care and care delivery. Our study found that innovations during the hospital discharge decision making is evolving as the pandemic persists and some changes were perceived as positive by rehabilitation clinicians.

Rehabilitation clinicians perceived many of the innovations in response to external stressors (pandemic) as having potential positive or negative impacts on patient outcomes. These findings highlight the need for future research to evaluate the impact of the following perceived innovations identified by participants to determine the patient, clinician, and system-level outcomes: increased ease of team collaboration due to virtual options, changes in caseload prioritization in the hospital setting, increased consideration of transitions of care directly to the home, and the increased availability and accessibility of virtual care in the home. Many of these innovations align with prioritization of aging in place” which is known to have many benefits from patients [40, 41]. It may be that clinicians and systems saw firsthand the benefits of aging in place and perceived the risks of discharging home were not as high as previously assumed. Identification and evaluation of unintended consequences that are not deliberate or foreseen are needed to fully understand the impact innovations can have on multiple outcomes [42]. For example, a shift toward a “Rehab in Place” model of care may have implications for staffing structures and patient perceptions of care. There is also a need to recognize the toll of the pandemic on rehabilitation clinicians’ mental health and collective trauma from providing care during the pandemic [43]. Staff stress and burnout—despite innovations—is a necessary factor to consider when potentially redefining the role of rehabilitation clinicians in the hospital setting.

Our findings also highlight the importance of implementation frameworks—such as i-PARIHS—in identifying factors that hinder or facilitate innovations that improve health service delivery and patient outcomes. We applied the i-PARIHS framework in a novel way to identify organic clinical innovation developed during hospital discharge decisions when the system was stressed by the COVID-19 pandemic. While i-PARIHS is traditionally used to identify barriers and facilitators to implementation of a specified innovation, we used the framework to identify emerging innovations in response to the pandemic. Additionally, we used i-PARIHS to understand and describe the underlying context and recipient factors that potentially drove these innovations. Such an application of i-PARIHS allows future research to consider alternative approaches to evaluating emerging innovations that have the potential to translate knowledge into practice. Breton and colleagues utilized a similar approach, although not with i-PARIHS, to qualitatively examine how the pandemic modified the organization and processes of primary care and how those were implemented for continuous quality improvement [44]. Identifying and understanding these factors can inform future studies along the research continuum from effectiveness to implementation trials.

Our study’s strength is that it provides insight into evolving recipient and contextual factors that fostered innovation and contributes to the data to knowledge part of a LHS cycle. With a LHS lens, we applied the i-PARIHS framework in a novel way to identify organic clinical innovation developed during hospital discharge decisions when the system was stressed (in this case by the COVID-19 pandemic). The application of this method transcends disciplines, settings, communities, and systems as the need to iteratively evaluate and continuously learn is crucial to healthcare transformation and the overall vision of optimizing patient and population outcomes.

### Limitations

A few limitations of this study should be noted. First, the participants were recruited from and provided care in the VA health care system and, thus, the results may not be immediately transferrable to other non-VA health care systems. The COVID-19 pandemic created a natural experiment where all health care systems were stressed and, thus, responses to such stress may be similar across systems. In addition, we choose the VA given its vision to implement LHS principles and the infrastructure it is creating to support this vision. Second, due to union stipulations we were unable to record interviews or collect demographic data on participants other than disciplines and years of clinical experience. To address this, we used member checking to ensure we accurately captured the data. In terms of demographic data, the American Physical Therapy Association’s most recent data to date on members (2016–2017) indicates a majority identify as female, white, non-Hispanic with most having at or above a doctorate level terminal degree [45]. 

## Conclusions

Understanding the experiences of rehabilitation clinicians providing care and engaging in hospital discharge decisions prior to the pandemic and during the COVID-19 era is essential to informing real-time resource needs and promising approaches that can be adapted, implemented, evaluated, and scaled quickly in response to system stressors. Importantly, the information gathered by this qualitative study provides foundational data for future research and LHS efforts focused on improving transitions from the hospital to the next level care. The experiences captured by rehabilitation clinicians suggest responses to the ongoing pandemic and future, unforeseen stressors can be innovative and potentially a positive change to the current status quo.

## Supplementary Information

Supplementary Material 1.

## Data Availability

The datasets generated and/or analyzed during the current study are not publicly available to minimize identification of participants but may be available from the corresponding author on reasonable request.

## Abbreviations

VA: Veteran Affairs
VHA: Veteran Health Administration
LHS: Learning Healthy System
i-PARIHS: integrated Promoting Action on Research Implementation in Health Services
OT: Occupational Therapist
OTA: Occupational Therapist Assistant
PT: Physical Therapist
PTA: Physical Therapist Assistant
MVAHCS: Minneapolis Veteran Affairs Health Care System
